# Optimised Method for Short-Chain Fatty Acid Profiling of Bovine Milk and Serum

**DOI:** 10.3390/molecules27020436

**Published:** 2022-01-10

**Authors:** Cheng Li, Zhiqian Liu, Carolyn Bath, Leah Marett, Jennie Pryce, Simone Rochfort

**Affiliations:** 1 Agriculture Victoria Research, AgriBio, 5 Ring Road, Bundoora, VIC 3083, Australia; Cheng.li@agriculture.vic.gov.au (C.L.); Carolyn.Bath@agriculture.vic.gov.au (C.B.); Jennie.Pryce@agriculture.vic.gov.au (J.P.); Simone.rochfort@agriculture.vic.gov.au (S.R.); 2 School of Applied Systems Biology, La Trobe University, Bundoora, VIC 3083, Australia; 3 Agriculture Victoria Research, Ellinbank Centre, Ellinbank, VIC 3821, Australia; Leah.Marett@agriculture.vic.gov.au; 4 Centre for Agricultural Innovation, School of Agriculture and Food, Faculty of Veterinary and Agricultural Sciences, The University of Melbourne, Melbourne, VIC 3010, Australia

**Keywords:** bovine milk, serum, short-chain fatty acids, liquid chromatography-mass spectrometry

## Abstract

Short-chain fatty acids (SCFA, C2-C5) in milk and serum are derived from rumen bacterial fermentation and, thus, have the potential to be used as biomarkers for the health status of dairy cows. Currently, there is no comprehensive and validated method that can be used to analyse all SCFAs in both bovine serum and milk. This paper reports an optimised protocol, combining 3-nitrophenylhydrazine (3-NPH) derivatisation and liquid chromatography-mass spectrometry (LC-MS) analysis for quantification of SCFA and β-hydroxybutyric acid (BHBA) in both bovine milk and bovine serum. This method is sensitive (limit of detection (LOD) ≤ 0.1 µmol/L of bovine milk and serum), accurate (recovery 84–115% for most analytes) and reproducible (relative standard deviation (RSD) for repeated analyses below 7% for most measurements) with a short sample preparation step. The application of this method to samples collected from a small cohort of animals allowed us to reveal a large variation in SCFA concentration between serum and milk and across different animals as well as the strong correlation of some SCFAs between milk and serum samples.

## 1. Introduction

Bovine milk and serum lipidomic studies are important for the dairy industry, as some of the lipids of milk and serum are associated with the physical properties and nutritional value of dairy products, and they have potential value for predicting the health status and fertility of dairy cows [1,2]. Some lipids in milk are transferred from the blood, while others are from *de novo* synthesis in the mammary gland [3]. Blood lipids are derived from diet, adipose tissue metabolism, ruminal biohydrogenation and ruminal microorganism degradation [3]. In addition to triglycerides and phospholipids, blood and milk contain non-esterified or free fatty acids (FA), including short-chain FA (SCFA, C2-C5) or volatile FA, which are the products of ruminal bacterial activity.

The SCFA produced by microbial fermentation of plant cellulose in the rumen have multiple functions. For example, C2, C3 and C4 are precursors for *de novo* synthesis of FA in the mammary gland [4]. C3 is also an important precursor for gluconeogenesis, particularly in the liver, in order to provide energy for cellular function [5]. Moreover, ruminal SCFA are important signalling molecules, regulating a variety of physiological functions of the rumen [6]. However, the mechanisms for how milk and serum SCFA are regulated are not well understood. Diet appears to have a large influence on this as it can alter the rumen microbiome, the SCFA profile produced by these microorganisms and even subsequent changes in ruminal pH, which can affect SCFA absorption in blood [7,8]. During negative energy balance, the concentration of valeric acid (C5) in milk is reduced, possibly due to a redirection of propionate (C3) components to the synthesis of lactose [9]. In addition, heat stress (along with reduced feed intake) results in reduced *de novo* FA, including some SCFAs in milk [10].

Being closely linked to rumen microbiota activity and animal physiology, SCFAs have the potential to be used as an indicator for animal health. Indeed, an SCFA derivative β-hydroxybutyric acid (BHBA) is the clinical biomarker for the diagnosis of ketosis. Due to the invasiveness of blood collection, biomarkers associated with metabolic diseases and fertility-using milk metabolites (e.g., BHBA and free FA) are currently sought for with some success [2].

Various gas chromatography-mass spectrometry (GC-MS) and LC-MS methods have been reported for the analysis of SCFA in biological samples, especially human and mouse faecal and blood samples [11,12,13,14,15,16]. GC-MS methods generally involve a time-consuming sample preparation procedure, while some of LC-MS methods did not include acetic and propionic acids [11,12], which are the main end products of rumen microbial fermentation. Reports on SCFA analysis in milk are scarce. A GC method for analysing bovine milk SCFA was reported with good sensitivity, precision and recovery, but this method had long sample preparation and instrument processing times, and acetic acid and propionic acids were not measured [17]. Milk is a complex matrix, containing a large number of lipid classes and thousands of lipid species with free FA being only a minor fraction [18]. Consequently, methods that have been validated only with human serum samples may not be applicable to milk samples of dairy cows.

This paper reports method development and validation for the quantification of SCFA (acetic acid, propionic acid, isobutyric acid, butyric acid, 2-methylbutyric acid, isovaleric acid and valeric acid) in both bovine milk and serum samples. BHBA and hexanoic acid are also included in this study because of their relevance to animal health. By optimising derivatisation parameters and reversed phase LC-MS (RP-LC-MS) analysis, we show that our method is simple and reliable for SCFA analysis in both milk and serum samples. The applicability of the method in analysing other free FA (C8-C18) was also evaluated.

## 2. Materials and Methods

### 2.1. Milk and Serum Samples

Raw milk and serum were collected from healthy Holstein cows (age: 4–7 years) from the Department of Jobs, Precincts and Regions research farm, located in Ellinbank, Victoria, Australia. Mature raw milk samples (days in milk between 50 and 110) were collected from morning milking (an aliquot of the entire milk of a cow); blood samples were also collected in the morning from the tail vein and then they were allowed to clot for a minimum of 1 h before centrifugation to separate the serum. Both milk and serum samples were kept on ice before being transported to the laboratory and were stored at −80 °C. The time delay between sample collection and freezing was below 4 h. The experiment received animal ethics approval from the Agricultural Research and Extension Animal Ethics Committee of the Department of Economic Development, Jobs, Transports and Resources, Victoria, Australia. All methods were performed by approved staff members in accordance with the relevant standard operating procedures approved by the above-mentioned ethics committee.

### 2.2. Chemicals

SCFA standard mix (containing 1 mg/mL each of acetic acid, propionic acid, isobutyric acid, butyric acid, 2-methylbutyric acid, isovaleric acid and valeric acid in free acid form; product number: 47058); hexanoic acid (in free acid from) and BHBA standard; and derivatisation reagents 3-nitrophenylhydrazine hydrochloride (3-NPH·HCl), 1-ethyl-3-(3-dimethylaminopropyl) carbodiimide (EDC) and pyridine were purchased from Sigma-Aldrich. Solvents used for sample preparation and mobile phase were of chromatographic grade and were from Fisher Scientific (acetonitrile, 0.1% formic acid in water and 0.1% formic acid in acetonitrile).

### 2.3. SCFA Derivatisation

All derivatisation reagents and SCFA standards were prepared in acetonitrile/water (2:1, *v/v*). Raw milk and serum samples were allowed to thaw thoroughly at room temperature, and SCFAs in raw milk and serum were extracted by adding two volumes of pure acetonitrile in one volume of milk/serum, followed by vortex for 2 min and centrifugation for 15 min (13,000× *g*); the supernatant was used for SCFA measurement. SCFAs were derivatised based on the protocol for carboxylic acid analysis described by Han et al. [19] with modifications. Briefly, the derivatisation reaction was conducted in a 2-millilitre Eppendorf tube, placed in a water bath (30 °C) for 30 min with frequent shaking after sequentially adding 50 µL of 50 mM 3-NPH·HCl, 50 mM EDC and 7% pyridine to 100 µL of standards or samples. When the reaction was completed, 250 µL of Milli-Q water was added to each reaction mixture before LC-MS analysis.

### 2.4. LC-MS Conditions

All derivatised SCFAs were separated by an Atlantis PREMIER BEH C18 AX column (2.1 × 150 mm, 1.7 µm, Waters) on a Vanquish UPLC system (Thermo Scientific, Waltham, MA, USA) with sample tray and column compartments that are correspondingly maintained at 15 °C and 55 °C. Mobile phase consisted of 0.1% formic acid in water (A) and 0.1% formic acid in acetonitrile (B), and gradient elution was conducted by increasing mobile phase B from 5 to 50% in 20 min and then 100% in the next 3 min. Flow rate and injection volume were 0.2 mL/min and 5 µL, respectively.

Derivatised SCFAs were detected by an LTQ-Orbitrap Elite mass spectrometer (Thermo Scientific, Waltham, MA, USA) with a heated electrospray ionisation source. Capillary temperature and probe heater temperature were 300 °C, and the sheath, auxiliary and sweep gases were at 30, 10 and 0 units, respectively. The mass spectrometer was operated in positive (4.2 kV) ionisation mode with a full scan (120–1200 *m*/*z*) at 60,000 resolution (FTMS mode). SCFAs were identified using Xcalibur (Thermo Fisher Scientific) based on retention time and accurate mass matching. The quantification of SCFA was performed using external calibration curves. 

### 2.5. Method Validation

In order to determine the LOD, limit of quantification (LOQ) and linear range, a stock solution of standard mixture (0.1 mg/mL) was used to prepare nine dilutions with concentrations ranging from 0.001 to 10 µg/mL. Upon derivatisation, each concentration of standard mix was injected three times. The LOD, LOQ (both expressed in µmol/L) and linear range were determined as described previously [20].

In order to test the reproducibility or precision of the method, three raw milk samples and a bulked serum sample (from 6 dairy cows) were analysed 5 times (with 5 separate derivatisation reactions). Reproducibility was estimated by calculating mean concentrations, standard deviation (SD) and RSD values of the 5 measurements for each sample.

For the recovery experiment, two standard mixtures (0.33 µg/mL or 2.8~5.5 µmol/L and 3.3 µg/mL or 28~55 µmol/L, respectively, close to LOQ and the highest concentration of linear range) were spiked with respect to raw milk and serum samples before SCFA extraction (by acetonitrile) and derivatisation. Recovery (%) was calculated by using the following formula.

(1) $$\mathrm{Recovery}\;(\%)=\frac{\mathrm{amount}\;\mathrm{of}\;\mathrm{SCFA}\;\mathrm{standards}\;\mathrm{spiked}}{\mathrm{amount}\;\mathrm{of}\;\mathrm{total}\;\mathrm{SCFA}\;\mathrm{in}\;\mathrm{spiked}\;\mathrm{samples}-\;\mathrm{amount}\;\mathrm{of}\;\mathrm{SCFA}\;\mathrm{in}\;\mathrm{the}\;\mathrm{matrix}}\;\times \;100$$

In order to ascertain the reliability of our method, all validation parameters (LOD and LOQ determination, measurement reproducibility and spike-recovery test) were analysed at least 3 times, and one set of data is presented.

### 2.6. Method Application

A total of 20 raw milk and 20 serum samples collected from 20 dairy cows on the same day were analysed by using the current method. The concentration range of each SCFA in milk and serum samples was measured and compared; correlations among SCFA within each sample type as well as between raw milk and serum were also examined.

## 3. Results

**Figure 1:**
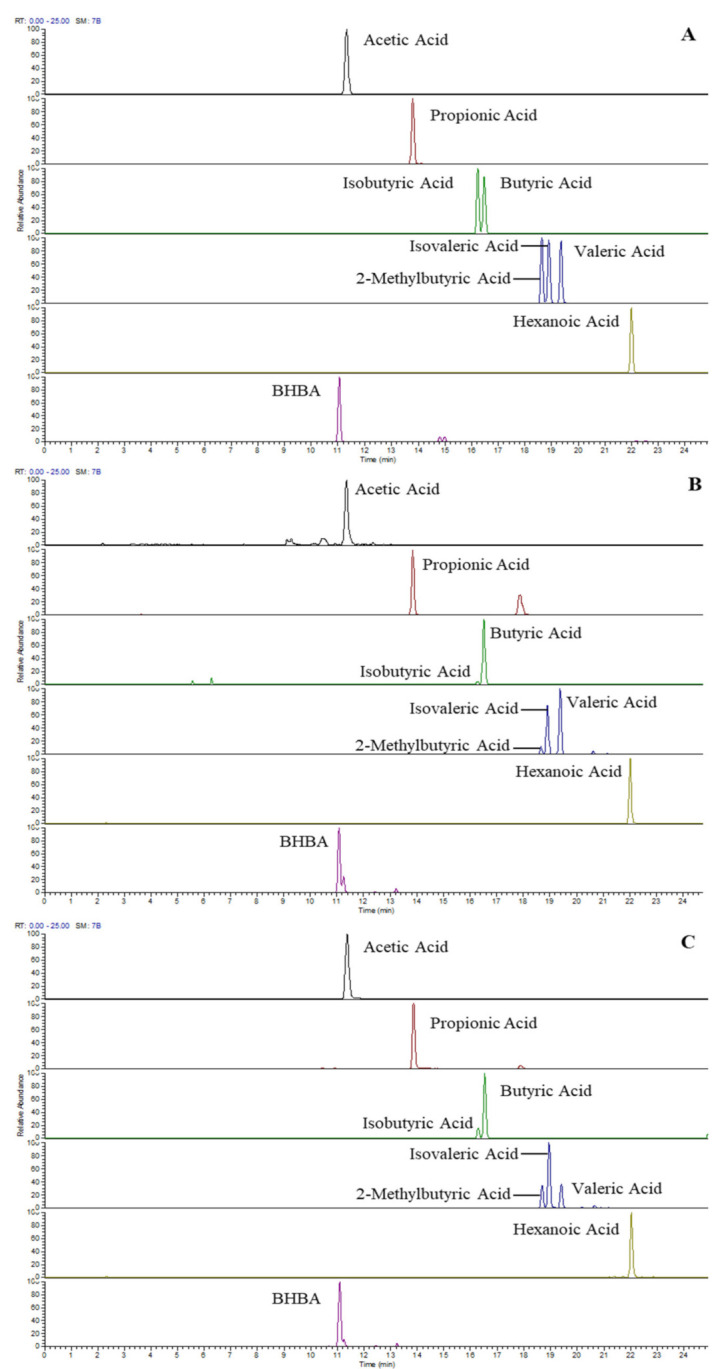
LC-MS profile (extracted ion chromatogram) of SCFA and BHBA acquired in positive ionisation mode from standards (3.3 µg/mL each or 28~55 µmol/L) (**A**), non-spiked raw milk (**B**) and non-spiked serum (**C**).

Baseline separations of C4/C5 isomers and satisfactory peak shapes of 3-NPH-derivatised SCFA were achieved by a 30-minute RP-LC run. Figure 1 shows the LC-MS profile (extracted ion chromatograms) of eight SCFAs and BHBAs from a standard mix (3.3 µg/mL each or 28~55 µmol/L) (A) and endogenous SCFA extracted from raw milk (B) and serum (C). Clearly, C4/C5 isomer composition differs between milk and serum samples, and all species are separated either by chromatography or by accurate mass difference. Table 1 lists the accurate mass, LOD, LOQ and linear range of derivatised SCFA under current sample preparation and LC-MS conditions. The LOD and LOQ for most SCFA is around 0.03 and 0.1 µmol/L, and the linear range is between 0.1 and 45 µmol/L. It is to be noted that acetic acid, hexanoic acid and BHBA show slightly lower sensitivity with this method.

The current method shows satisfactory reproducibility for measuring SCFA; the RSD values for repeated analyses are below 7% for all but one (isovaleric acid in one of the milk samples) and below 5% for most measurements (Table 2). It is worth noting that the larger RSD values are often associated with low concentrations of SCFA in samples (e.g., isovaleric acid in milk and hexanoic acid in serum). The overall analytical precision is similar across milk and serum samples, although there are net differences in RSD values between milk and serum samples for the same SCFA species (e.g., propionic acid and valeric acid showing a larger RSD with milk).

When SCFA standards were spiked at two concentrations (0.33 µg/mL each or 2.8~5.5 µmol/L and 3.3 µg/mL each or 28~55 µmol/L) into milk and serum, the recovery was 84-115% for most analytes. A lower, yet acceptable, recovery rate (74–80%) was observed with acetic acid at the low spike level (Table 3). These results indicate that the current sample preparation protocol is reliable for quantification of SCFA and BHBA in both raw milk and serum samples.

Optimised SCFA extraction and derivatisation methods were applied to a cohort of 20 healthy cows in order to determine the concentration range of each SCFA and BHBA in normal milk and serum samples. Erratic results were observed for one milk sample and two serum samples (probably due to contamination/lipolysis during sample handling); thus, these samples were excluded from analysis. Table 4 summarises the results obtained from the remaining 19 milk samples and 18 serum samples.

All SCFAs and BHBAs show large inter-cow variation (2-20-fold difference) in milk as well as serum. Overall, serum contained a greater concentration of acetic acid, propionic acid, isobutyric acid, 2-methylbutyric acid, isovaleric acid and BHBA but a lower concentration of butyric acid and hexanoic acid compared to milk (Table 4). Very low concentrations of valeric acid were found in both milk and serum.

When a pair-wise correlation analysis was performed using these data, a number of significant correlations (*r* > 0.8) were revealed across SCFAs within and between the two sample types (Table 5). In milk samples, the strongest correlation was observed between isobutyric acid and 2-methylbutyric acid and between butyric acid and hexanoic acid. In serum samples, in addition to the isobutyric acid/2-methylbutyric acid pair, a strong correlation was observed between butyric acid and isovaleric acid and between acetic acid and BHBA. Furthermore, a strong correlation was found between milk and serum samples for isobutyric acid and 2-methylbutyric acid.

## 4. Discussion and Conclusions

A simple and sensitive LC-MS method for quantification of SCFA in bovine milk and serum is potentially of great value for monitoring dairy cow health status and improving the quality of milk production. 

As direct analysis of SCFA by LC-MS suffers from low sensitivity, a derivatisation method using 3-NPH has been described with varying reagent concentrations, reaction temperatures and reaction times [12,19,21,22,23]. Methanol was used as the solvent for dissolving SCFA as well as for dissolving derivatising reagents in some studies [12,19]. However, we have found that, albeit affording excellent reaction yield, methanol (HPLC grade) contains a substantial amount of SCFA impurities (particularly acetic acid and propionic acid), affecting the accuracy of quantification. Among the alternative solvents we have tested (ethanol, isopropanol and acetonitrile), acetonitrile had the lowest amount of SCFA impurities and did not significantly affect the determination of LOD and LOQ; thus, acetonitrile was chosen as the solvent for extracting SCFA from milk and serum samples as well as the derivatisation reaction medium. Han et al. [19] reported that derivatisation of different carboxylic acids by 3-NPH can be conducted at 0 °C for 60 min or 30 °C for 30 min. We have found no significant differences in reaction yield between these two conditions (results not shown); thus, all derivatisation reactions in this study were performed at 30 °C for 30 min.

The LOD for most SCFA reaches 0.0033 µg/mL (around 0.03 µmol/L) after optimising sample preparation and LC-MS parameters, which is suitable for determining SCFA concentration in bovine milk and serum [24,25,26,27]. Since SCFAs are highly volatile, it is very challenging to enrich them in a liquid sample. Thus, method sensitivity is of great importance for SCFA quantification in raw milk and serum samples. The method offers an LOD of 0.1 µmol/L for BHBA, which is suitable for accurately monitoring the health of transition dairy cows, given that subclinical ketosis is defined as blood serum BHBA above a threshold of 1200 μmol/L [28]. The sample preparation procedure (SCFA extraction and derivatisation) of this method is suitable for SCFA quantification in both raw milk and serum, as evidenced by the low RSD (<7% for most analytes) of repeated analyses of three milk samples and a bulked serum sample. The method also affords adequate recovery for most SCFA (84–114%); a slightly lower recovery for low-level acetic acid implies that the method could slightly underestimate acetic acid when present at a very low concentration. This low recovery is believed to be associated with the high volatility of this SCFA species and inevitable loss during sample preparation.

Various LC-MS methods have been reported for analysing SCFA in human faeces and serum. To our knowledge, this is the first report on SCFA analysis in milk using LC-MS. When compared to a GC method for identification and quantification of SCFA in cow milk [17], our LC-MS method had higher sensitivity and shorter sample preparation and instrument running time. In addition, the method described in this study has the potential to be used for the simultaneous analysis of SCFA and medium and long-chain fatty acids (Figure S1, Supplementary Materials). 

The large inter-cow variation in SCFA content suggests that SCFA molecules may be closely associated with individual animal metabolism as all animals were managed in the same herd with the same feeding regime. The strong correlation between butyric acid and hexanoic acid in milk samples can be explained by their precursor–product relationship in *de novo* synthesis in the mammary gland. On the other hand, the correlation between isobutyric acid and 2-methylbutyric acid within and between milk and serum samples may result from their common origin of rumen bacterial activity and their circulation in intact form from the blood to mammary gland and then to milk. Serum SCFAs are generally used as biomarkers for the activity of rumen microbiota and health status of animals. The strong correlation of isobutyric acid and 2-methylbutyric acid between milk and serum suggests that these two SCFAs of milk may be potential biomarkers for rumen bacterial activity and animal health. The concentration of serum BHBA (ranging from 468 to 1372 µmol/L) was below 1200 µmol/L for the majority of the cows, indicating a scarcity in ketosis incidence across these lactating cows. 

In conclusion, a simple, sensitive and reliable LC-MS method was optimised, which enables BHBA and SCFA in raw milk and serum to be measured using the same protocol. Our preliminary survey using a small cohort of animals has demonstrated the possibility of using milk SCFA as biomarkers for the status of rumen microbiota. In addition, the current method has the potential to be used for the quantification of all free FAs in milk, which are known to be potential biomarkers of cow fertility.

## Figures and Tables

**Table 1:** Accurate mass, LOD, LOQ and linear range of derivatised SCFA and BHBA.

Name	Accurate Mass * (*m/z*)	LOD (µmol/L)	LOQ (µmol/L)	Linear Range (µmol/L)	R^2^
Acetic Acid	196.0722	0.17	0.55	0.55–166.61	0.9974
Propionic Acid	210.0879	0.0446	0.14	0.14–44.57	0.9999
Isobutyric Acid	224.1035	0.0375	0.11	0.11–37.48	1.0000
Butyric Acid	224.1035	0.0375	0.11	0.11–37.48	0.9997
2-Methylbutyric Acid	238.1192	0.0323	0.10	0.10–32.33	0.9999
Isovaleric Acid	238.1192	0.0323	0.10	0.10–32.33	0.9999
Valeric Acid	238.1192	0.0323	0.10	0.10–32.33	0.9999
Hexanoic Acid	252.1348	0.09	0.28	0.28–28.43	0.9993
BHBA	240.0984	0.10	0.32	0.32–96.11	0.9980

* Accurate mass of derivatised SCFA and BHBA in positive ionisation mode.

**Table 2:** Mean concentrations and SD (µmol/L), and RSD (%, *n* = 5) of derivatised SCFA and BHBA in three raw milk and one bulked serum samples.

SCFA	Milk									Serum		
	Sample 1			Sample 2			Sample 3			Bulked Sample		
	Mean	SD	RSD	Mean	SD	RSD	Mean	SD	RSD	Mean	SD	RSD
Acetic acid	12.00	0.373	3.11	12.530	0.667	5.32	14.62	0.879	6.02	1379.45	61.76	4.48
Propionic acid	1.52	0.104	6.84	1.813	0.122	6.74	2.33	0.091	3.89	30.93	0.82	2.64
Isobutyric acid	0.23	0.010	4.45	0.265	0.008	3.12	0.30	0.017	5.63	3.09	0.07	2.40
Butyric acid	67.82	2.205	3.25	69.822	3.204	4.59	128.87	2.383	1.85	21.65	0.93	4.28
2-Methyl-butyric acid	0.10	0.004	3.96	0.150	0.007	4.65	0.19	0.005	2.51	1.49	0.04	2.99
Isovaleric acid	0.13	0.009	6.83	0.151	0.010	6.41	0.16	0.013	8.24	1.94	0.06	3.22
Valeric acid	0.75	0.050	6.74	0.822	0.037	4.47	1.37	0.031	2.30	0.77	0.02	2.92
Hexanoic acid	32.30	1.506	4.66	35.342	1.922	5.44	64.07	1.690	2.64	1.90	0.08	4.17
BHBA	22.34	0.092	0.41	20.836	0.285	1.37	26.68	0.442	1.66	922.99	24.49	2.65

**Table 3:** Recovery (% ± SD, *n* = 3) of spiked SCFA from raw milk and serum matrices at two spike levels (Low: 0.33 µg/mL each and High: 3.3 µg/mL each).

Name	Raw Milk		Serum	
	Low	High	Low	High
Acetic Acid	74.3 ± 5.0	93.8 ± 0.7	79.7 ± 10.0	93.9 ± 4.0
Propionic Acid	97.4 ± 3.0	97.0 ± 0.6	112.9 ± 2.1	110.3 ± 3.1
Isobutyric Acid	100.6 ± 1.9	101.0 ± 1.1	110.3 ± 1.3	109.4 ± 1.5
Butyric Acid	98.1 ± 2.5	98.2 ± 0.5	110.5 ± 2.5	110.1 ± 1.8
2-Methylbutyric Acid	96.9 ± 1.0	98.3 ± 1.5	113.4 ± 1.5	110.9 ± 0.4
Isovaleric Acid	99.5 ± 1.2	104.2 ± 0.6	111.8 ± 1.1	110.6 ± 1.0
Valeric Acid	98.4 ± 0.9	102.4 ± 0.6	111.1 ± 2.2	111.5 ± 2.8
Hexanoic Acid	96.6 ± 2.5	98.6 ± 0.7	102.4 ± 1.3	111.4 ± 0.8
BHBA	100.4 ± 0.1	96.9 ± 1.0	84.1 ± 8.4	96.4 ± 2.2

**Table 4:** Concentration range (µmol/L) of BHBA and SCFA in raw milk (*n* = 19) and serum (*n* = 18).

Name	Raw Milk	Serum
Acetic Acid	4.33–20.83	815.38–1922.32
Propionic Acid	0.68–2.57	14.86–43.76
Isobutyric Acid	0–1.02	1.59–18.63
Butyric Acid	11.58–175.46	11.81–31.00
2-Methylbutyric Acid	0–0.39	0.88–11.95
Isovaleric Acid	0.20–0.39	1.27–3.43
Valeric Acid	0.39–1.96	0.10–0.78
Hexanoic Acid	4.82–58.06	0.09–1.72
BHBA	14.42–43.83	468.44–1372.64

**Table 5:** Pairwise correlation among SCFA and BHBA in raw milk (*n* = 19) and serum (*n* = 18).

		Raw Milk									Serum							
		C2	C3	Iso-C4	C4	2-MBA *	Iso-C5	C5	C6	BHBA	C2	C3	Iso-C4	C4	2-MBA	Iso-C5	C5	C6
Raw Milk	C3	0.24																
	Iso-C4	−0.09	0.26															
	C4	−0.21	0.05	0.34														
	2-MBA	−0.22	0.25	0.92	0.31													
	Iso-C5	0.10	0.67	0.26	0.10	0.34												
	C5	−0.35	0.37	0.36	0.57	0.49	0.61											
	C6	−0.17	0.10	0.28	0.98	0.28	0.14	0.55										
	BHBA	0.71	−0.03	0.13	−0.11	−0.08	−0.11	−0.46	−0.11									
Serum	C2	0.07	−0.37	0.03	−0.21	−0.05	−0.32	−0.43	−0.23	0.24								
	C3	−0.21	−0.18	0.13	−0.28	0.23	−0.01	0.02	−0.30	−0.25	0.59							
	Iso-C4	−0.30	−0.07	0.94	0.36	0.87	0.06	0.27	0.29	0.05	0.29	0.32						
	C4	−0.06	−0.16	0.12	−0.14	0.14	−0.13	−0.26	−0.15	0.03	0.46	0.40	0.22					
	2-MBA	−0.37	−0.01	0.91	0.35	0.95	0.17	0.41	0.29	−0.13	0.20	0.38	0.94	0.27				
	Iso-C5	−0.10	−0.12	0.20	−0.15	0.23	−0.07	−0.25	−0.14	0.10	0.51	0.30	0.27	0.88	0.35			
	C5	−0.10	−0.34	−0.23	−0.24	−0.04	−0.30	−0.14	−0.22	−0.18	0.25	0.54	−0.04	0.35	0.08	0.27		
	C6	0.18	−0.24	−0.27	−0.23	−0.31	−0.25	−0.43	−0.20	0.23	0.49	0.48	−0.05	0.00	−0.19	−0.12	0.30	
	BHBA	0.25	−0.39	−0.08	−0.09	−0.21	−0.42	−0.64	−0.08	0.50	0.82	0.16	0.14	0.49	0.03	0.59	0.20	0.41

* 2-MBA: 2-methylbutyric acid.

## Data Availability

Data are available from the authors.

## Supplementary Materials

The following is available online, Figure S1: LC-MS profile of derivatised medium and long-chain fatty acids acquired in positive ionisation mode from raw milk.
