# Supplementary material for: Optimised Method for Short-Chain Fatty Acid Profiling of Bovine Milk and Serum
Source: Molecules. 2022 Jan 10;27(2):436. doi: 10.3390/molecules27020436 (PMC8778335; doi:10.3390/molecules27020436)
Supplement: Supplementary file 1 [file molecules-27-00436-s001.zip › molecules-1481549-supplementary.pdf]

Supplementary Information:

Optimised method for short-chain fatty acid profiling of bovine milk and serum

Cheng Li[1,2], Zhiqian Liu[1], Carolyn Bath[1], Leah Marett[3,4], Jennie Pryce[1,2] and Simone Rochfort[1,2]

[1]Agriculture Victoria Research, AgriBio, 5 Ring Road, Bundoora, Victoria 3083, Australia

[2]School of Applied Systems Biology, La Trobe University, Bundoora, Victoria 3083, Australia

[3]Agriculture Victoria Research, Ellinbank Centre, Ellinbank, Victoria 3821, Australia

[4]Centre for Agricultural Innovation, School of Agriculture and Food, Faculty of Veterinary and Agricultural Sciences, The University of Melbourne, Victoria 3010, Australia

Corresponding author: Zhiqian.liu@agriculture.vic.gov.au

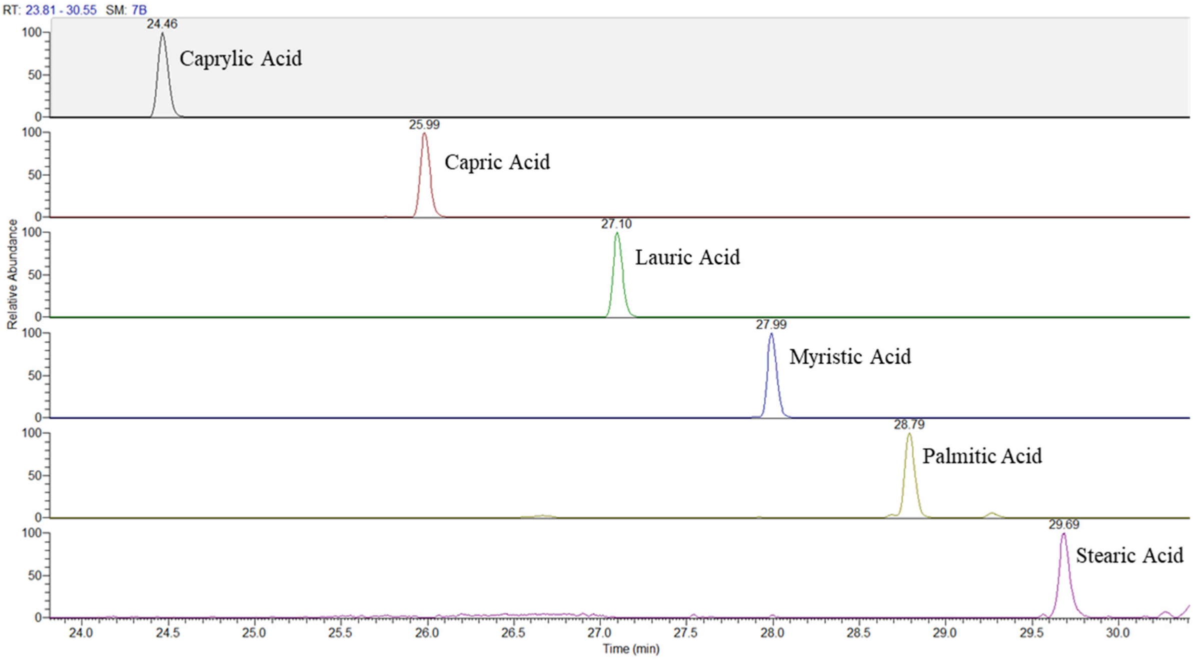

Figure S1. LC-MS profile of derivatised medium- and long-chain fatty acids acquired in positive ionisation mode from raw milk.
